# Supplementary material for: 4-Pyridoxic Acid/Pyridoxine Ratio in Patients with Type 2 Diabetes is Related to Global Cardiovascular Risk Scores
Source: Diagnostics (Basel). 2019 Mar 6;9(1):28. doi: 10.3390/diagnostics9010028 (PMC6468858; doi:10.3390/diagnostics9010028)
Supplement: Supplementary file 1 [file diagnostics-09-00028-s001.zip › diagnostics-445996 sp for final/Supplemental Figures.pdf]

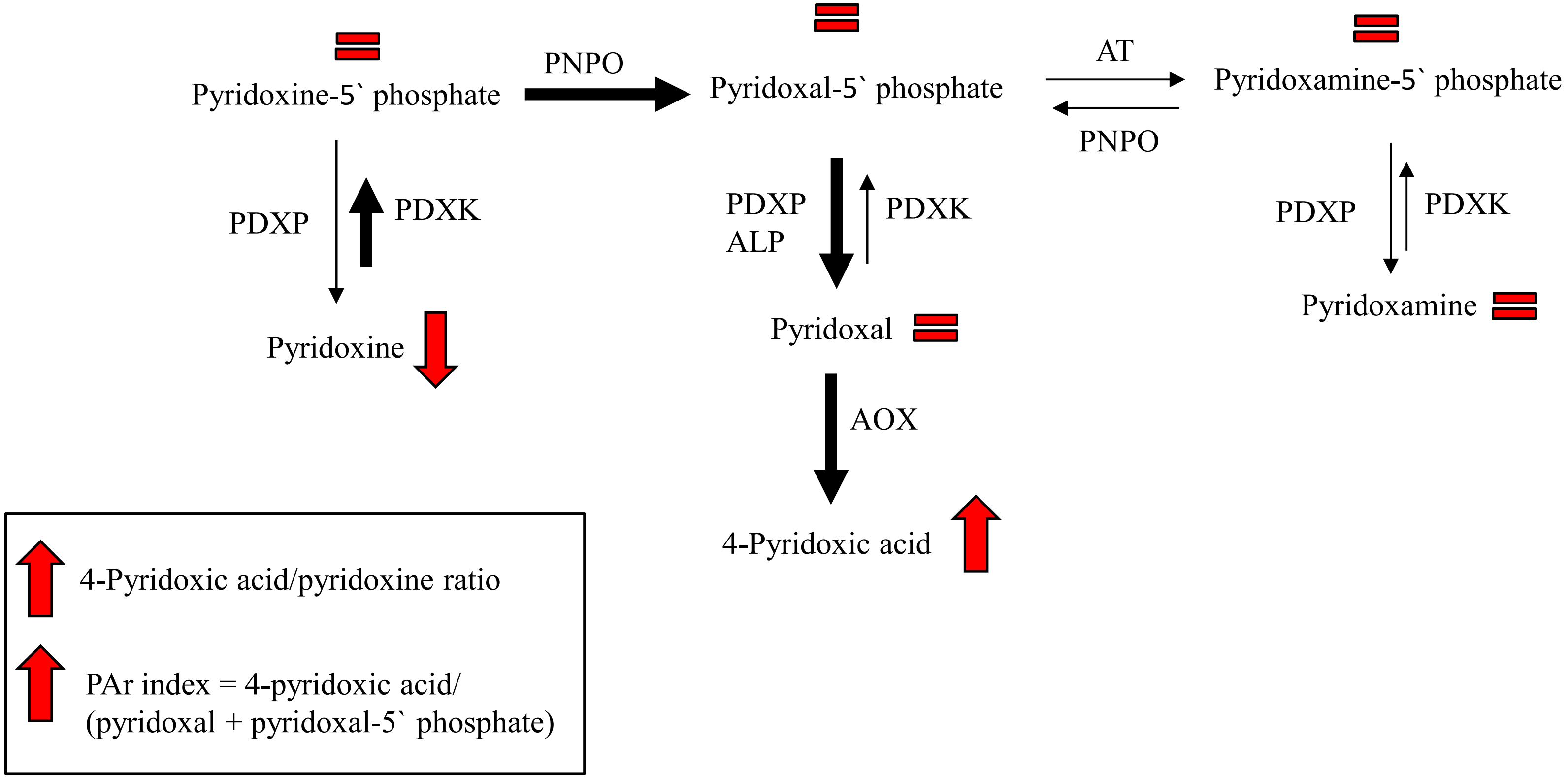

Supplemental Figure 1. Vitamin B6 catabolism and summary of the changes of plasma vitamin B6 vitamers associated with high cardiovascular risk scores in patients with type 2 diabetes. Advanced risk scores were associated with elevated 4-pyridoxic, lowered pyridoxine, and increased 4-pyridoxic acid/pyridoxine ratio and PAr index. The association of 4-pyridoxic acid/pyridoxine ratio with risk scores was independent on inflammation and renal function.

Abbreviations: AT, aminotransferase; PDXK, pyridoxal (or pyridoxine) kinase; PNPO, pyridoxamine 5-phosphate oxidase; ALP, alkaline phosphatase; PDXP, pyridoxal (pyridoxine) phosphatase; AOX, aldehyde oxidase.

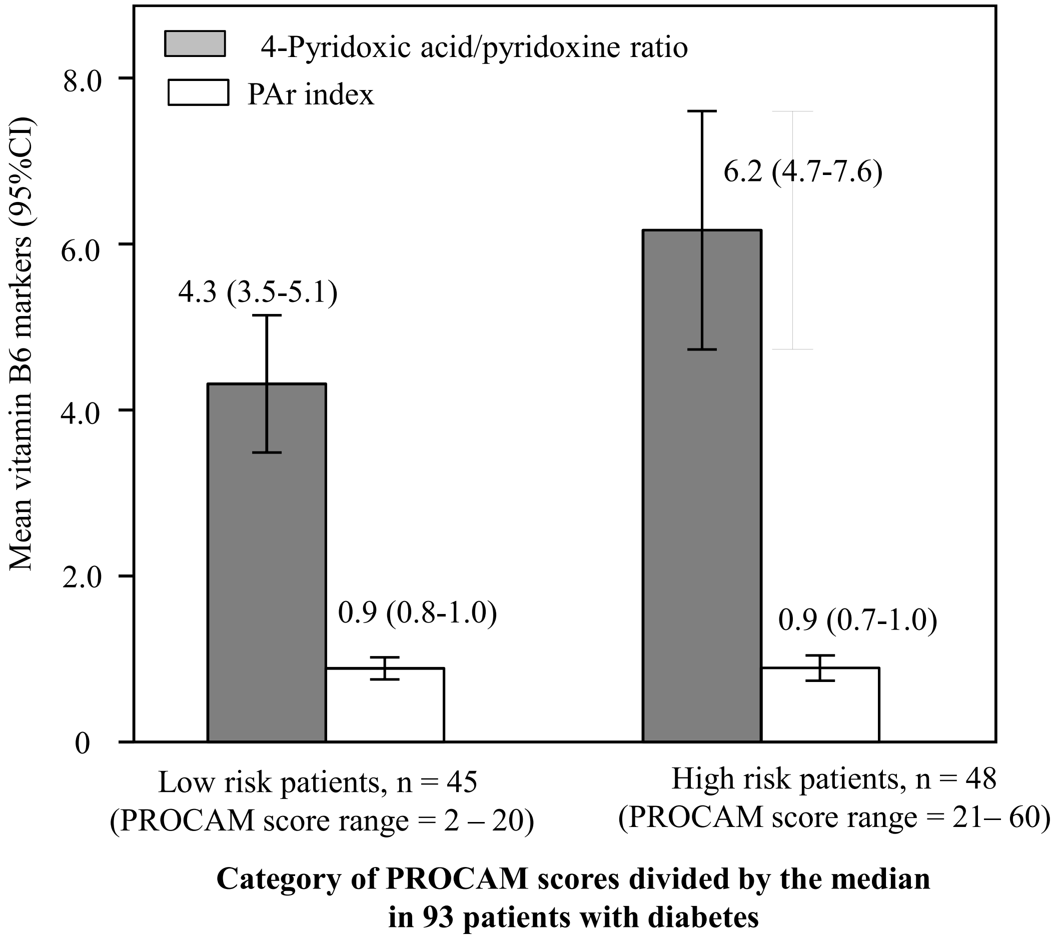

Supplemental Figure 2. Mean and 95% Confidence Intervals (CI) of 4-pyridoxic acid/pyridoxine ratio and PAr index [= 4-pyridoxic acid/(pyridoxal + pyridoxal-5` phosphate)] according to Prospective Cardiovascular Münster (PROCAM) study. PROCAM score relies on classical risk factors (LDL-C, HDL-C, triglycerides, smoking, diabetes, hypertension, age, and sex) to estimate the 10-y risk of cardiovascular major event. The difference in 4-pyridoxic acid/pyridoxine ratio between the low and the high PROCAM score categories: P = 0.030 (unadjusted); P = 0.046 (adjusted for cystatin-C and CRP). For PAr, 4-pyridoxic acid/pyridoxine ratio was not significantly different according to PROCAM score. 93 patients with diabetes (age up to 75 years) were separated by the median of PROCAM score into low and high risk groups.
