# Supplementary material for: 4-Pyridoxic Acid/Pyridoxine Ratio in Patients with Type 2 Diabetes is Related to Global Cardiovascular Risk Scores
Source: Diagnostics (Basel). 2019 Mar 6;9(1):28. doi: 10.3390/diagnostics9010028 (PMC6468858; doi:10.3390/diagnostics9010028)
Supplement: Supplementary file 1 [file diagnostics-09-00028-s001.zip › diagnostics-445996 sp for final/Supplemental tables.pdf]

Supplemental Tables

Supplemental Table 1. Risk scores for cardiovascular hospitalization in patients with type 2 diabetes based on common risk factors [see Yu et al (1) for details on the multivariate model].

Constant	-3.80246
Risk factor	***Beta Coefficient***
Sex (men = 1; women = 0)	0.2289
Age ≥ 70 years (1 = yes; 0 = no)	0.8159
HbA1c ≥ 7.4% (1 = yes; 0 = no)	-0.0397
(BMI/10)^-2	- 1.8538
(BMI/10)^0.5	+ 0.9606
(Systolic BP/100)$^{\wedge 2}$	-0.4030
(Systolic BP/100)$^{\wedge 2}$* Ln(systolic BP/100)	0.9662
(Diastolic BP/100)$^{\wedge 2}$	0.474
(Diastolic BP/100)$^{\wedge 2}$* Ln(diastolic BP/100)	0.2724
Ln(total cholesterol/10)	0.5147
(Total cholesterol/10)^0.5	-1.0580
Ln(HDL-C)	0.0734
(HDL-C/10)^3	-0.0238
(LDL-C/10)^0.5	-0.5563
ln(LDL-C/10)*(LDL-C/10)^0.5	-0.8316

The risk for cardiovascular hospitalization = 1/ (1+e $-^{\text{risk score}}$).

Plasma concentrations of lipids were entered in SI (mmol/L) units.

BMI, body mass index; BP, blood pressure; HbA1c, glycated hemoglobin; HDL-C, high density lipoprotein cholesterol; LDL-C, low density lipoprotein cholesterol.

Supplemental Table 2. Traditional risk factors in the healthy subjects compared to the patients with type 2 diabetes.

	All patients N = 122	Control subjects N = 34	p
Men, n (%)	60 (50%)	24 (29%)	0.033
Age, years	69.9 (9.1)	49 (9)	<0.001
BMI, kg/m^2	29.8 (4.1)	25.1 (4.2)	<0.001
Systolic BP, mmHg	138 (15)	124 (13)	<0.001
Diastolic BP, mmHg	82 (7)	80 (7)	0.254
Cystatin-C, µg/ml	1.03 (0.46)	0.35 (1.03)	<0.001
CRP, µg/ml	5.7 (12.5)	2.2 (4.6)	0.120
sVCAM-1, ng/ml	531 (236)	411 (138)	0.005
Total cholesterol, mg/dl	197 (37)	222 (34)	0.001
HDL-C, mg/dl	49 (14)	65 (19)	<0.001
LDL-C, mg/dl	125 (34)	142 (28)	0.009
Triglycerides, mg/dl	155 (81)	95 (37)	<0.001
Framingham risk scores	35.2 (11.7)	7.9 (5.5)	<0.001
PROCAM score	60.3 (11.4)	28.4 (10.4)	<0.001

Data are mean (SD).

P values are according to ANOVA test (log transformed values).

BMI, body mass index; BP, blood pressure; CRP, C-reactive protein; HDL-C, high density lipoprotein cholesterol; LDL-C, low density lipoprotein cholesterol; sVCAM-1, soluble vascular cell adhesion molecule 1.

Supplemental Table 3. Correlations of PAr and 4-pyridoxic acid/pyridoxine ratio with single risk factors (only continuous variables) in 122 patients with type 2 diabetes.

Variable	PAr index	4-Pyridoxic acid/pyridoxine ratio
Age	0.191 (p = 0.035)	0.212 (p = 0.021)
Creatinine	0.187 (p = 0.040)	NS
Cystatin -C	0.215 (p = 0.017)	NS
CRP	0.285 (p = 0.001)	0.151 (p = 0.101)
Risk score from Yu et al (1)	0.173 (p = 0.057)	0.299 (p = 0.001)
PROCAM score	NS	0.193 (p = 0.035)
sVCAM-1	NS	0.383 (p < 0.001)

The correlation coefficients are according to Spearman test. Only significant correlations or those with a trend are shown. PAr index = 4-pyridoxic acid/ (pyridoxal + pyridoxal-5`phosphate) ratio. No correlations were observed between PAr or 4-Pyridoxic acid/pyridoxine ratio and other risk factors such as systolic and diastolic blood pressure or blood lipids.

Supplemental Table 4. Plasma vitamin B6 forms in healthy controls (n = 34) compared to patients with diabetes and low vascular risk (n=61)

Vitamin B6 forms	Patients with diabetes and low risk N=61	Control subjects N = 34	p
4-Pyridoxic acid, nmol/L	31.9 (15.0)	29.7 (40.6)	0.649
Pyridoxine, nmol/L	38.1 (127.8)	13.6 (5.4)	0.462
Pyridoxal, nmol/L	13.9 (5.7)	25.0 (18.0)	<0.001
Pyridoxal-5`phosphate, nmol/L	29.3 (16.2)	55.9 (72.3)	0.007
Pyridoxamine, nmol/L	1.8 (1.0)	3.2 (13.9)	0.476
Pyridoxamine phosphate, nmol/L	10.0 (5.1)	5.5 (11.5)	0.010
4-Pyridoxic acid/pyridoxine ratio	3.9 (3.2)	2.2 (2.5)	0.009
PAr index	0.84 (0.06)	0.37 (0.12)	<0.001

Data are mean (SD).

P values are according to ANOVA test (log transformed values).

PAr index = 4-pyridoxic acid/ (pyridoxal + pyridoxal-5`phosphate) ratio.

Reference

1. Yu D, Cai Y, Graffy J, Holman D, Zhao Z, Simmons D. Development and external validation of risk scores for cardiovascular hospitalisation and rehospitalisation in diabetes patients. J Clin Endocrinol Metab 2018; 103:1122-9.
